# The Potential of Fatty Acids and Their Derivatives as Antifungal Agents: A Review

**DOI:** 10.3390/toxins14030188

**Published:** 2022-03-03

**Authors:** Ana Guimarães, Armando Venâncio

**Affiliations:** 1 Centre of Biological Engineering, University of Minho, 4710-057 Braga, Portugal; avenan@deb.uminho.pt; 2 LABBELS—Associate Laboratory, 4710-057 Braga, Portugal

**Keywords:** fungal contamination, mycotoxins, oxylipins, hydroxy fatty acid, unsaturated fatty acids, saturated fatty acids

## Abstract

Fungal contamination presents several problems: in humans, health issues arise from infections with opportunistic filamentous fungi and yeast, while in food, fungi cause spoilage and, in particular, in the case of mycotoxigenic fungi, can cause serious health issues. Several types of fatty acids and their derivatives, oxylipins, have been found to have inhibitory effect towards fungal growth and the production of mycotoxins. The use of fatty acids as antifungals could fulfil consumer’s requests of more natural and environmentally friendly compounds, while being less likely to promote fungal resistance. In addition, due to their nature, fatty acids are easily used as food additives. In this work, we review the most relevant and recent studies on the antifungal ability of fatty acids. We focused on saturated fatty acids, unsaturated fatty acids, and oxylipins, their different impact on fungal inhibition, their proposed modes of action, and their ability to impair mycotoxin production. Applications of fatty acids as antifungals and their limitations are also addressed.

## 1. Introduction

**Figure 1:**
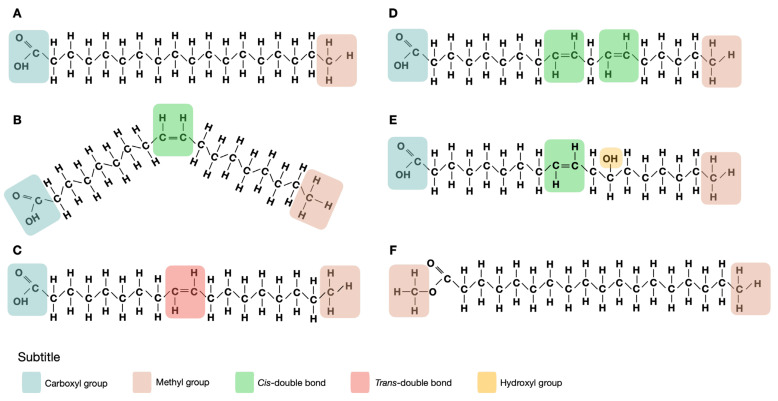
Chemical structure of fatty acids and derivatives: (**A**) saturated fatty acids (stearic acid); (**B**) cis monounsaturated fatty acid (cis oleic acid); (**C**)trans monounsaturated fatty acid (trans oleic acid); (**D**) polyunsaturated fatty acid (linoleic acid); (**E**) hydroxy fatty acid (ricinoleic acid); (**F**) fatty acid methyl ester (stearic acid methyl ester).

Fatty acids are the main components of lipids. They are mainly found in the form of triacylglycerols, where three fatty acids are attached to a glycerol molecule. Different fatty acids at different positions in the glycerol molecule influence its properties (such as digestibility and melting point) [1]. Additionally, phospholipids are also a significant source of fatty acids. Fatty acids are organic acids that are characterized by having a carboxyl group (–COOH) and a methyl group (–CH3). Naturally occurring fatty acids can be classified by the number of double bonds present in the aliphatic chain: saturated fatty acids (SFA), when no double bonds are present; monounsaturated fatty acids (MUFA), when there is a single double bond; and polyunsaturated fatty acids (PUFA), when there are two or more double bonds present. Double bonds can be arranged in cis or trans configurations, where both hydrogen atoms are on the same side of the double bond or on opposite sides of the fatty acid, respectively. The chemical structures of different types of fatty acids are shown in Figure 1. Cis configuration is more common in nature and is the predominant arrangement found in foods, while *trans* configuration is less common, and is typically found in small quantities in fat of ruminant animals and in milk, or by partial hydrogenation of unsaturated oils [2].

Fatty acids can additionally be classified by their carbon chain length and the position of the first double bond on the carbon chain from the methyl terminal [3]. PUFAs are classified as omega 6 (*n*-6) and omega 3 (*n*-3), when their first double bound is between the 6th and 7th carbon atoms or between the 3rd and 4th carbon atoms, respectively, when counting from the methyl group. Most of these molecules are formed by a straight carbon chain, since they are synthetized from two-carbon units, the chain length can extend from 2 to 80 carbon atoms, even though most common fatty acids have 12 to 22 carbon atoms. However, fatty acids with odd carbon numbers (such as heptadecanoic acid), with branched chains (e.g., isopalmitic acid) or carbocyclic units (e.g., sterculic acid) also occur naturally [4].

Fatty acids are ubiquitous in nature and have a crucial role in several life processes. Some of the most significant roles of fatty acids are structural, as constituents of phospholipids; as part of neutral lipids, which serve as cell energy storage; and as signaling compounds in several pathways. Furthermore, fatty acids from animal fat, plants, and other sources are an essential part of human and animal nutrition [5].

Despite the difficulty in detecting associations between diet components and the occurrence of either beneficial or detrimental health effects, the consumption of certain fatty acids has been strongly connected with several health benefits. Replacing saturated fatty acids with MUFA or PUFA reduces low-density-lipoprotein (LDL) cholesterol, thus reducing the risk of cardiovascular disease. The ingestion of unsaturated fatty acids is thought to be associated with a reduced risk of several cancers, and a number of inflammatory conditions (such as Crohn’s disease, arthritis, and asthma) could also be, potentially, improved by the increase consumption of these fatty acids [1]. On the other hand, trans fatty acids, especially those produced by partial hydrogenation, are reported to have numerous adverse health effects including cardiovascular disease, inflammation, oxidative stress, increase in LDL cholesterol, increase in body weight, insulin sensitivity, and cancer; without providing nutritional benefits [6]. Consequently, several expert committees and governing bodies emitted statements limiting *trans* fatty acids intake [7,8,9].

Fatty acid derivatives, such as oxygenated derivatives named oxylipins, have also crucial importance in all biological systems. They take part in development-regulated processes and as a response to environmental changes. In plants, oxylipins take part in the crosstalk between pathogens and their host. Plant oxylipins are produced by the oxidation of unsaturated fatty acids via several metabolic pathways and include fatty acid peroxides, hydroxy-, oxo-, and keto-fatty acids, divinyl ethers, volatile aldehydes, and jasmonic acid. These participate actively in plant defense mechanisms, namely plant defense signaling pathways, and are thought to be essential for the resistance of plants to pathogens [10].

For decades the antifungal properties of fatty acids have been under the attention of researchers, driven by the necessity to find substances that avoid fungal resistance and that have reduced environmental risk. Research papers from as early as 1945 describe the successful inhibition of both filamentous fungi and yeasts by fatty acids and related compounds, specially long-chain fatty acids [11]. Work regarding the use of fatty acids as antifungals continues to be relevant with recent papers evaluating the use of fatty acids in new delivery systems, such as electrospun patches and nanoparticles, to inhibit pathogenic fungal growth [12,13]. Despite the interest of fatty acids as antifungal agents, very few reviews have addressed this topic [14].

Here, we review the role of fatty acids and their derivatives, especially oxylipins, as inhibitory agents of fungal growth and, also, mycotoxin synthesis. We focused on fungal infection in crop plants (especially those used in human nutrition) and fungal contamination of food products. Still, we also include examples of antifungal fatty acids for clinic uses.

## 2. The Problem of Fungal Contamination

Fungi are eukaryotic, multinucleated organisms, with heterotrophic nature, which have a chitinous cell wall and can present a filamentous growth in multicellular colonies. These organisms are ubiquitous, being able to occupy a vast range of environments, such as soil, water, air, and several other materials [15]. It is estimated that nearly 1.5 to 5.1 million types of fungi exist, but only approximately 120,000 species have been identified [16].

Fungi present several problems, from health issues and animal diseases to crop losses and food spoilage. Some species of filamentous fungi and yeasts are opportunistic pathogens that can cause infections and contribute significantly to human morbidity and mortality [17]. Systemic fungal infections have increased in the last decades, mainly due to the increase in population with abnormal host defenses against infections. Regarding the most relevant health problems that arise from opportunistic fungal infections, *Candida* spp. cause oral, lower intestinal track, and vaginal infections [18]; *Cryptococcus neoformans* and *Cryptococcus gatti* are known to infect immunocompromised people [19], and *Aspergillus* spp. are responsible for aspergillosis, an opportunistic lung infection caused by the inhalation of airborne spores and can also cause otomycosis [20,21]. Recently, mucormycosis, an infection caused by the filamentous of the *Mucorales* order that have a mortality rate of 50%, have been opportunistically infecting COVID-19 patients [22]. Overall, over 600 different fungi have been reported to infect humans, mainly mucosa, skin, hair, and nails, or cause allergies [17].

Fungi may also cause major problems at any stage of the food processing chain (i.e., in the field, during post-harvest, processing, storage, and handling steps) since they are able to grow in diverse environmental conditions [23,24,25,26]. Fungal proliferation in crops and foods causes severe losses in commodities, high economic deficits, and several health risks, due to the toxicity and pathogenicity of some species [27]. It is estimated that more than 600 million people could be fed each year if this contamination could be mitigated in the five most important crops [28], and with the predictable population growth, food loss represents, at the same time, a waste of resources and an environmental problem.

A part of the health risks caused by fungal contamination is also due to the production of mycotoxins by some filamentous species, which occur naturally in agriculture products and processed foods [29]. Mycotoxins are small and stable molecules, extremely difficult to degrade. Additionally, they can persist and accumulate in organisms when the exposure is chronic [30]. Castillo et al. (2016) reports that there are approximately 600 mycotoxins, with new toxins still being discovered, which brings additional concerns and implications to food safety [31]. It is difficult to assess the impact of mycotoxins; still, it is believed that 25% of the world’s agricultural commodities are contaminated [32]. The improvement of food safety is a major concern, leading worldwide organizations to establish regulations to reduce mycotoxins exposure [33,34], since these toxins can cause severe adverse health effects in humans, including carcinogenic, mutagenic, teratogenic, neurotoxic, nephrotoxic, immunosuppressive, and estrogenic effects, even when ingested at low concentrations [35].

Due to all the negative effects of fungal contamination, several strategies have been developed to prevent fungal growth. Strategies for the prevention of fungal and mycotoxin contamination can be applied during pre-harvest, harvest, and post-harvest. Despite the numerous methods available to deal with the fungal contamination problem, many of them have disadvantages. Preventive measures directed at the inhibition of fungi proliferation in agricultural products are the most efficient approach to avoid the presence of fungi and toxins in the food chain. Nowadays, the most common food preservation strategies involve chemical or physical techniques, which may only decrease fungal infection, but eventually alter the characteristics of food and feed. In addition, the use of synthetic antifungals has led to the development of resistance, which has led to the need for greater concentrations, thus increasing toxic residues in food products, and also having a negative impact in the environment. In addition, consumer’s trends are moving towards minimally processed, green-labelled, and “chemical free” food products [36].

The use of fatty acids as antifungals can have several advantages when compared with synthetic chemicals. They are natural and environmental friendly; fulfil consumer’s expectation for the use of less synthetic chemicals as food preservatives; can be used to control plant disease, reducing chemicals that negatively affect the environment; and are less likely to promote resistance in fungi [37]. They can also be recovered from waste materials, thus favoring sustainability by promoting a circular economy [38].

**Figure 2:**
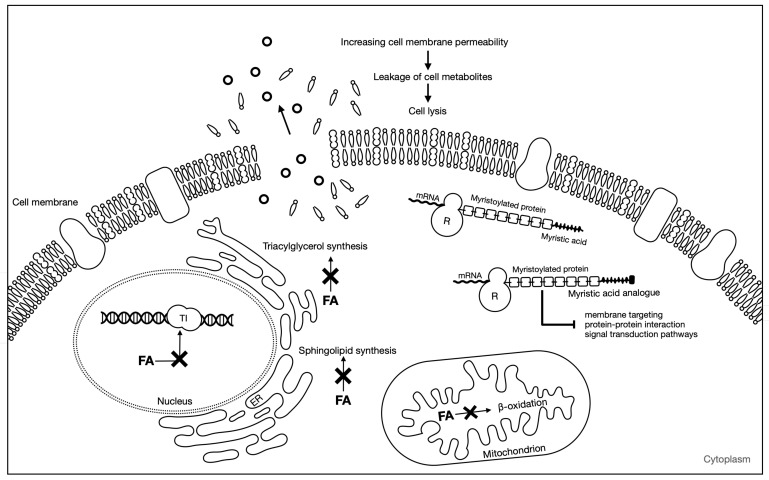
Antifungal mechanisms of fatty acids. FA: fatty acid; TI: topoisomerase I; ER: endoplasmic reticulum; R: ribosome. Adapted from Pohl et al. [14].

The main proposed mechanism of action of antifungal fatty acids states that fatty acids insert themselves into the lipid bilayers of fungal membranes compromising membrane integrity, resulting in an uncontrolled release of intracellular electrolytes and proteins, eventually leading to cytoplasmatic disintegration of fungal cells [39]. Chemical composition and environmental pH are especially important in the antifungal ability of these compounds. Thibane et al. (2012) proposed that several PUFAs may increase the unsaturation ratio of the cellular membrane, leading to the accumulation of intracellular reactive oxygen species (ROS) and a consequent loss of mitochondrial membrane potential [40]. However, other mechanisms may also be responsible for the antifungal activity of fatty acids. For instance, fatty acids are able to inhibit topoisomerase I [41,42], an enzyme involved in the breaking and repairing of DNA strands and in topological changes necessary for cellular process such as replication, transcription, and recombination [43]. Cis-monounsaturated fatty acids are the most efficient in inhibiting topoisomerase I; with geometry, the position of the double bond and the carbon chain length of the fatty acids influences this inhibitory process [41]. However, very few studies have been conducted on fatty acids ability to inhibit fungal topoisomerases [14]. Fatty acid biosynthesis inhibition can also be a mechanism responsible for the antifungal ability of fatty acids. For instance, 2-hexadecynic acid has been found to interfere with the elongation of saturated and unsaturated fatty acids, and it is thought to inhibit triacylglycerol synthesis [44] and 6-nonadecynoic acid was suggested to act by interfering with sphingolipid biosynthesis [45,46]. Other mode of action of fatty acids is through inhibition of the enzyme N-myristoyltransferase (NMT). NMT catalyzes the reaction of myristoylation, in which myristic acid takes part. This reaction plays a key role in membrane targeting, protein–protein interaction, and signal transduction pathways. Several myristic acid analogues have been shown to inhibit NMT in vitro. The incorporation of these analogues into fungal cells competes with myristic acid binding to NMT and interrupts the reaction of myristoylation. Ultimately, this leads to disruption of protein function resulting in fungal growth inhibition [47]. A scheme representing the main mechanisms of fatty acids antifungal activity is shown in Figure 2.

The use of fatty acids and their derivatives as antifungals could be a solution for some fungal contaminations, either applied directly or in a perspective of biopreservation when produced by beneficial microorganisms, such as lactic acid bacteria. This would be of particular interest in the case of fermented food products, such as cheese, sausages, and sourdough.

## 3. Fatty Acids and Derivatives as Antifungal Agents

### 3.1. Saturated Fatty Acids

Saturated fatty acids have been reported to exert inhibitory activity towards some fungal species. Hydrophobic groups of SFA seem to play a role in the antimicrobial activity [48]. However, long chain fatty acids have a reduced solubility in aqueous environments, due to the high number of hydrophobic groups, while shorter chain fatty acids may not have enough hydrophobic groups to interact with the cell membrane. Lauric acid (C12:0) has been found to have the best balance of hydrophobic and hydrophilic groups, when considering antimicrobial activity [48,49].

Capric acid (C10:0) has been reported to have cytotoxicity against *Candida albicans* at 10 mM, leading to the disruption or disintegration of the plasma membrane, due to changes in intracellular hydrostatic turgor pressure. The same study described the activity of lauric acid, which was effective at a 2.5 mM concentration [50]. Similar results have been previously reported by Kabara et al. (1977), which showed that capric, lauric, and palmitoleic acids were able to inhibit *C. albicans* [49]. More recently, and also studying the inhibition of *C. albicans*, Lee et al. (2021) demonstrated that medium chain saturated fatty acids (C7:0, C8:0, C9:0, C10:0, C11:0, and C12:0) exhibited antimicrobial activity with Minimum Inhibitory Concentration (MIC) from 100 to 200 μg/mL and biofilm formation inhibition by 75% at 2 μg/mL. These fatty acids were also shown to mimic the quorum sense molecule farnesol, and thus interfere with this system, leading to inhibition of biofilm and hyphal formation [51]. Several other authors reported the effect of saturated fatty acids in *C. albicans* [52,53]. More recently, Bhattacharyya et al. (2020), used computational simulations to analyze the interaction of caprylic acid with a model lipid bilayer cell membrane. In silico results determined that caprylic acid was able to interact with and penetrate the model membrane. Transmission electron microscopy showed that short exposure of *Malassezia furfur* to 0.2% caprylic acid resulted in disarrayed granular cytoplasm. In vitro studies showed that caprylic acid ester derivative, propylene glycol monocaprylate, had potent membrane disruptive actions against *C. albicans* and *M. furfur* and that the combination of this derivative with azole antifungals showed an increased effect in azole resistant *C. albicans* [54]. Capric and pelargonic (or nonanoic) acids were found to have inhibitory effects against the dermatophyte *Micosporum gypseum* with MIC ranging from 0.02 to 75 μg/mL and 40 to 50 μg/mL, for capric acid and pelargonic acid, respectively [55].

In their study, Liu et al. (2008) tested butyric, caproic, caprylic, capric, lauric, myristic, and palmitic acids against four economical important phytopathogenic fungi (*Alternaria solani*, *Colletotrichum lagenarium*, *Fusarium oxysporum* f. sp. *Cucumerinum*, and *F. oxysporum* f. sp. *Lycopersici*). Results showed that all the fatty acids inhibited mycelial growth and, except for myristic acid, SFA also inhibited spore germination [56]. Aneja et al. (2005) reported that nonanoic acid, produced by *Trichoderma harzianum*, was able to inhibit spore germination and mycelial growth of cocoa pathogens, *Crinipellis perniciosa* Stahel and *Moniliophthora roreri* Cif. H.C. Evans. Inhibition reached 75% for both spore germination and mycelial growth, with mycelial growth being less sensible to nonanoic acid. Authors concluded that *Trichoderma* spp. could be used in a biocontrol strategy in cocoa plantations [57]. Decanoic and octadecanoic acids have been found to be toxic to fermenting yeasts, in conjugation with ethanol, leading to the interruption of grape must fermentation [58]. Regarding the use of saturated fatty acids in food applications, lauric, myristic, and palmitic acids were evaluated for their activity against fungi isolated from cheese surfaces (*F. oxysporum* and *F. avenaceum*). Lauric acid inhibited *F. oxysporum* completely at 20 μg/mL; myristic and palmitic acids reduced the radial growth rate of both fungi at 40 μg/mL, but their effect was reversible [59]. Furthermore, Corsetti et al. (1998) discovered that a *Lactobacillus sanfrancisco* strain produced caproic acid (C6:0), which contributed to the antifungal effect of this strain against molds responsible for bread spoilage [60].

Despite the existing reports of antifungal activity of saturated fatty acids, most studies reporting this activity have been published decades ago, and most of the recent studies have focused on the far more active unsaturated fatty acids.

### 3.2. Unsaturated Fatty Acids

Inhibitory action of unsaturated fatty acids is considerably higher than that of saturated ones, partially because of the presence of double bonds that occupy a greater cross section, thus creating increased motional freedom in the target organism membrane [39]. It is generally considered that PUFAs owe their antimicrobial properties to their structure and shape, such as chain length and the number, position, and orientation of double bounds. Fatty acids with double bonds with cis configuration were shown to be stronger antimicrobials than the ones with trans double bonds, due to the lower thermodynamic stability of cis double bonds when compared to trans. Thus, *cis* bounds are thought to induce greater membrane deformations on the targeted microorganisms [61,62]. Unsaturated medium and long chain fatty acids are considered safe, regarded as environmentally “green”, and are currently used in animal feed to enrich meat with health-beneficial properties [63].

Several works describe the antifungal ability of free unsaturated fatty acids. Sensitivity to fatty acids was associated to a high degree of unsaturation of phospholipid fatty acids and low proportion of sterol [64]. Stearidonic acid (18:4 *n*-3), eicosapentaenoic acid (20:5 *n*-3), docosapentaenoic acid (22:5 *n*-3), and docosahexaenoic acid (22:6 *n*-3) have been found to have inhibitory effect on the mitochondrial metabolism and biofilm formation of *C. albicans* and *Candida dublinienses*, thus being useful in the treatment of *Candida* biofilms, which present increased antifungal resistance [65].

Walter and co-works [66] studied the antifungal ability of four unsaturated fatty acids (linoleic, linolenic, oleic, and erucic acids) against plant pathogenic fungi, and verified that linolenic and linoleic acids at a concentration of 1000 μM reduced the mycelial growth of the all the fungi in study (*Rhizoctonia solani*, *Pythium ultimum*, *Pyrenophora avenae,* and *C. perniciosa*), with linoleic and linolenic acid reaching inhibitions greater than 55%. Oleic acid at 1000 μM could only reduce growth by 35% and erucic acid had no effect on fungal growth. *Cis*-9-heptadecanoic acid was found to inhibit mycelial growth and conidial germination of several phytopathogenic fungi at a concentration of 150 μg/mL. Events leading to fungi sensitivity to this fatty acid were, sequentially: (i) partitioning of the fatty acid into fungal membranes, (ii) increase in membrane fluidity, dependent of sterol content, leading to higher membrane disorder, causing conformational changes in membrane proteins, and (iii) ultimately, cytoplasmatic disintegration [39]. Unsaturated fatty acids were also related to the growth inhibition of *Cladosporium cucumerinun* and *F. oxysporum* (by 51 and 33%, respectively). PUFAs also play an important role in plant–microbe interactions, either as free fatty acids or as their oxygenated derivatives. For instance, linoleic acid levels take part in the regulation of the development, seed colonization and mycotoxin production by *Aspergillus* spp. [67]. Additionally, increased levels of this fatty acid induce greater resistance to attack by some pathogenic fungi (*Colletotrichum gloeosporioides*) [68]. However, there are also reports that linoleic acid stimulates the production of spores in several fungal species (such as *Alternaria tomato*, *Sclerotinia fruticola,* and *Neurospora crassa*). It is suggested that fatty acids might regulate fungal development by mimicking or interfering with signals that regulate fungal sporogenesis [67].

### 3.3. Oxylipins

**Figure 3:**
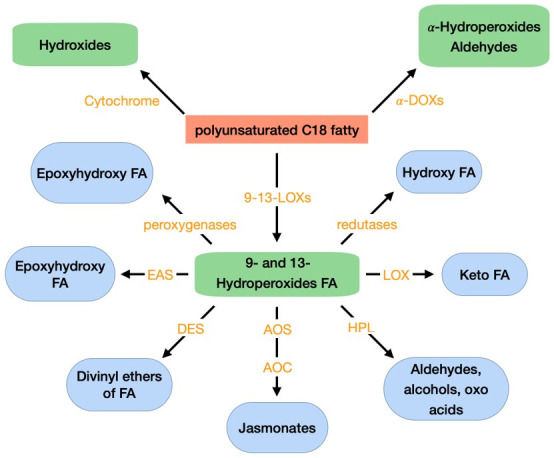
Oxidative metabolism of PUFA C18 fatty acids in plants. FA: fatty acid; LOX: lipoxygenase; α-DOX: α-dioxygenase; AOC: allene oxide cyclase; AOS: allene oxide synthase; DES: divinyl ether synthase; EAS: epoxy alcohol synthase; HPL: hydroperoxide lyase. Adapted from Deboever et al. (2020) [69].

Oxylipins are a vast family of secondary metabolites originated from the oxidation and further conversions of PUFAs, mainly linoleic acid and linolenic acid; but also, hexadecatrienoic acid, arachidonic acid, eicosapentaenoic acid, and docasahexaenoic acid. These compounds are found in almost all organisms and can exist in free forms, esterified to phospholipids or galactolipids, or combined with other molecules [69]. Oxylipins are crucial for several biological activities as signals of intra- and inter-cellular communication in plants, vertebrates, invertebrates, and fungi [70]. In fungi, oxylipins are implicated in the regulation of cell development, asexual and sexual spore development, adaptive responses and production of secondary metabolites [70]. In plants, oxylipins play a role in plant defense mechanisms against pathogens and pests, activating stress signaling pathways. These compounds can also act as molecular signals to regulate plant growth and development, senescence, sex determination of reproductive organs, programmed cell death, can have antimicrobial activity and act as quorum-sensing molecules [70,71,72,73]. The family of plant oxylipins and of the enzymes that originate them can be seen in Figure 3. Oxylipins biosynthesis from PUFAs, in plants, are predominantly initiated by a lipoxygenase (LOX) and a cyclooxigenase (COX)—α-dioxygenase (α-DOX). Several authors describe how the reduction in LOX resulted in increased susceptibility of plants to pathogens [74], while overexpression increased resistance to pathogens [75,76]. LOX has been shown to catalyze the formation of hydroperoxides from PUFAs and transformed them into monohydroxy PUFA, which can be additionally oxidized to ketones. Oxylipins can also be formed in non-enzymatic pathways through free radical mediated oxidation of ROS. Reactions mediated by ROS can convert PUFAs into hydrogen peroxides. These can be, then, reduced to monohydroxy fatty acids. There is increasing evidence that oxylipins formed without enzymatic aid, also, play important role in plant stress responses [77,78].

The family of plant oxylipins is very versatile in its chemical structure, with many geometric isomers. Position isomers of the same oxylipins can have different biological activity, especially when alterations in the hydroxy or epoxy groups occur [79]. Moreover, oxylipins originated from linolenic acid seem to be more effective, regarding pathogen inhibition than the ones originated from linoleic acid [72].

However, the mechanisms of antimicrobial activity of oxylipins remains unclear. From different studies, it appears that the primary target of oxylipins is the cell membrane [39]. The inhibitory activity of oxylipins in fungal or bacterial growth measured in vitro, in some studies, seems to be transitory, which indicates that oxylipins may not affect cell viability and only delay growth [72]. However, in spore germination assays, long-term inhibition of germination occurs, indicating that the presence of oxylipins might result in spore death. It was proposed that several oxylipins might induce plant cell damage and defense gene expression through a mechanism based on their electrophilic nature. Still, it is thought that oxylipins can also affect membrane permeability (pore formation and membrane destabilization). Although oxylipins have reduced hydrophobicity, when compared to fatty acids, thus decreasing their ability to permeate membranes, they may be still able to cause the disruption of fungal membranes. There is also the possibility of oxylipins being able to interfere with microbial metabolism or signaling. This occurs by competing with structurally similar endogenous lipids that cause protein denaturation or oxidative bursts [69,72].

The mechanisms of this antimicrobial effects should be further elucidated by multidisciplinary approaches (such as proteomics, metabolomics, and transcriptomics) that will be fundamental to the establishment of oxylipins as new biocontrol agents. Additionally, similarities in plant oxylipins and fungal oxylipins, suggest that fungal oxylipins might be able to take control of the host oxylipin pathway to facilitate disease development and the production of spores and mycotoxins [69].

Based on this literature search, oxylipins might have great potential as biocontrol agents; however, the mechanisms underlying oxylipins action are still mainly unknown. Oxylipins play a significant role in plant protection against pathogens, either by defense responses or by having direct antifungal activity, and since oxylipins occur naturally in plants used for human consumption, the regulatory requirements for their use could be reduce.

#### 3.3.1. Hydroxy Fatty Acids

Among the most studied oxylipins regarding antifungal ability are hydroxy fatty acids [80,81,82]. Hydroxy fatty acids have a hydroxy group in the carbon chain. The presence of this group alters its properties, increasing viscosity and reactivity compared to non-hydroxy fatty acids [83]. In addition to their antifungal ability, hydroxy fatty acids have been connected to other biological functions, such as signaling, virulence, and response mechanisms towards stress factors [84].

The antifungal activity of hydroxy fatty acids is thought to be related to specific structural attributes, being dependent on the number of double bounds and the presence of hydroxy groups in the middle of the carbon chain. Antifungal activity varies with the number and position of the hydroxy groups [83]. Unsaturated hydroxy fatty acids with hydroxylation at the C9–C13 positions in a C18 chain, showed significant antifungal activity while C2 and C18-OH analogues were less active. This might be related with differences in dissociation constants or different interaction with the fungal membrane [64,85]. Unsaturation is thus fundamental for the activity of long chain hydroxy fatty acids, but not for middle-chain hydroxy fatty acids. Moreover, the position of double bonds and chirality seems to have only minor impact on antifungal ability [72,86].

The antifungal ability of hydroxy fatty acids is probably linked to their detergent-like interaction with cell membranes that alters membrane fluidity [87]. Both hydroxy and non-hydroxy fatty acids diffuse rapidly into membranes without assistance of membrane proteins [87], thus not explaining the usually stronger antifungal ability of hydroxy fatty acids. Studies also determine that there were no differences in membrane polarity when fungal membranes were subjected to treatments with fatty acids and hydroxy fatty acids. So, differences in antifungal ability between hydroxy fatty acids and non-hydroxy fatty acids may relay in the targeting of specific regions or lipids rafts of the fungal membrane [85]. Additionally, in phospholipids bilayers, there is a preference for intercellular fatty acid binding proteins towards hydroxy fatty acids instead of their non-hydroxy precursors [88]. All these factors contribute for the increased antifungal activity of hydroxy fatty acids compared to their non-hydroxy forms.

Besides structural attributes of hydroxy fatty acid molecules, their antifungal ability also seems to be related to fungal physiology. (R)-8-hydroxy-cis-9,cis-12-octadecadienoic acid is the precocious-sexual-inducer factor that regulates the sexual development of *Aspergillus* spp.; additionally, this compound is involved in antifungal activity against phycomycetous fungi, regulation of conidia formation, and production of mycotoxins [89]. All these functions of hydroxy fatty acids in fungal physiology and ecology demonstrate that their modes of action might be species specific [80]. In addition, some studies indicate that fungal sensitivity towards fatty acids may be related to low fungal sterol content [39]. Sterol is a membrane-fluidity modifier that maintain the structure of the membrane during stress [90]. The same is observable for hydroxy fatty acids, with studies showing that fungi with high sterol content were resistant to hydroxy fatty acids, while fungi with lower sterol content were sensitive to these compounds [85].

Liang et al. (2017) tested hydroxy unsaturated fatty acids as antifungal agents against both filamentous fungi and yeasts and concluded that the inhibitory action of hydroxy fatty acids targeted filamentous fungi (including food borne pathogens such as *Penicillium roqueforti* and *Aspergillus niger*), whereas tested yeasts (food fermentations species and the pathogen *C. albicans*) showed resistance to these fatty acids. These results could make hydroxy unsaturated fatty acids a viable antifungal for fermented products that require growth and activity of yeast. In another study, coriolic (13-hydroxy-9,11-octadecadienoic) acid and rinoleic (12-hydroxy-9-octadecenoic) acid were tested against important phytopathogens. In vitro results indicated that both acids had strong activities regarding *Leptosphaeria maculans* and *A. niger*, with activity varying with different fungal species [80]. On wheat and barley, only coriolic acid reduced the severity of disease caused by *Pyrenophora* spp., but, at high concentrations, coriolic acid and rinoleic acid had toxic effects in some plants, indicating that further evaluation is needed before applying these hydroxy fatty acids in crop protection [91]. Furthermore, Liang et al. (2020) reported that hydroxy unsaturated fatty acids with a hydroxyl group in the center of an 18-carbon fatty chain displayed stronger antifungal activity. Since the most sensitive fungi tested had a lower content of ergosterol, authors propose that hydroxy fatty acids susceptibility is possibly related with ergosterol content of the cytoplasmatic membrane [92]. Still, the inhibitory activity of hydroxy fatty acids towards food spoilage organisms is considerably unexplored and the resistance mechanism of yeasts and filamentous fungi remain unexplained.

Since lactic acid bacteria (LAB) can convert linoleic and linolenic acids into hydroxy fatty acids, many works that report hydroxy fatty acid’s antifungal ability use these bacteria for their production. The use of LAB in this context could be particularly beneficial for the control of food-borne fungi in foods fermented by these bacteria.

Sjögren et al. (2003) were the first to report the antifungal activity of hydroxy fatty acids produced by LAB. In their work, several 3-hydroxy fatty acids (3-(R)-hydroxydecanoic acid, 3-hydroxy-5-cis-dodecenoic acid, 3-(R)-hydroxydodecanoic acid, and 3-(R)-hydroxytetradecanoic acid) were isolated from *Lactobacillus plantarum* MiLAB 14 supernatant and their broad antifungal activity was demonstrated against several molds and yeasts. In this case, medium chain hydroxy fatty acids appear to inhibit yeast more efficiently than filamentous fungi [82]. Black et al. (2013) verified that the addition of linoleic acid to the culture medium of *Lactobacillus hammesi* DMS 16381 resulted in an increase in the production of coriolic acid and ricinoleic acid. Both hydroxy fatty acids demonstrated antifungal activity against *A. niger*, with a MIC of 700 μg/mL and 2400 μg/mL, respectively. The use of sourdough fermented with *L. hammesi* DMS 16381 was able to delay the growth of *A. niger* and *P. roqueforti* and increase bread shelf life for up to 3 days. Coriolic acid at 0.15% was as effective as 0.4% calcium propionate in bread against *P. roqueforti* and environmental contaminants [81].

Ndagano et al. (2011) reported that *L. plantarum* and *Weissella paramesenteroides* strains, isolated from cassava, showed antifungal activity against *Aspergillus* and *Penicillium* species and that this activity was attributed to the production of 2-hydroxy-4-methylpentatoic acid and phenyllactic acid, in a synergy with other metabolites [93]. Broberg et al. (2007) also detected high levels of two hydroxy fatty acids (3-hydroxydecanoic acid and 2-hydroxy-4-methylpentanoic acid) in silages inoculated with *L. plantarum* strains that exerted antifungal activity in combination with other antifungal compounds [94]. In addition, Brosnan et al. (2012) detected six fatty acids, including 3-hydroxydecanoic acid and DL-β-hydroxymyristic acid, in the supernatant of a LAB strain that demonstrated antifungal ability [95].

In general, hydroxy fatty acids have a strong antifungal activity against a large number of fungi. A compilation of works that describe the use of hydroxy fatty acids and their MICs is shown in Table 1. Some works report MICs ranging from 10 to 100 μg/mL, which are similar with common antifungal drugs (such as amphotericin B) [82]. However, the conversion of linoleic and linolenic acids into antifungal hydroxy fatty acids by bacteria, may vary in situ. Therefore, more studies are needed to elucidate and improve the production of antifungal hydroxy fatty acids in different food matrixes.

#### 3.3.2. Acetylenic Fatty Acids

Acetylenic fatty acids are identified by a carbon triple bond. As with other oxylipins, acetylenic fatty acids are produced by plants when attacked by fungi as a defense mechanism [14]. Acetylenic fatty acids, as 2-alkynoic fatty acids, have been described as being toxic for fungi. The antifungal activity of these fatty acids is dependent on the chain length and environmental pH. Carbon chain lengths between 8 and 16 carbons have been defined for 2-alkynoic fatty acids to have maximum antifungal effects [99].

The 2-hexadecynoic acid has been studied more thoroughly for its antifungal, antimicrobial, and cytotoxic properties, which have been linked to its ability to inhibit the elongation and acylation process (particularly triacylglycerol synthesis) of fatty acids [100]. In addition, 6-nonadecynoic acid, isolated from the roots of *Pentagonia gigantifolia*, was found to have antifungal properties against *C. albicans* strains with MIC values (0.52 μg/mL), similar to commercial antifungals amphotericin B (0.52 μg/mL) and fluconazole (0.29 μg/mL). Authors suggested that 6-nonadecynoic acid interferes with fungal sphingolipid biosynthesis [46]. This fatty acid was also found to have activity against *C. neoformans*; however, it was deemed inactive towards other strains of *C. albicans* [101]. The 6-nonadecynoic acid and octadecynoic acid were found to be active against *Candida krusei* [56]. Chemically synthetized 2,6-hexadecadiynoic acid demonstrated antifungal activity towards fluconazole-resistant *C. albicans* strains (MIC of 11 μM), and against *C. neoformans* (MIC of <5.7 μM) [102]. The 9,11,13-octadecatriynoic acids were found to have activity against the plant pathogens *F. oxysporum*, *Magnaporthe oryzae*, *Phytophthora capsica*, *Phytophthora infestans*, *R. solani,* and *Sclerotiniai sclerotiorum* [103]. These compounds could have potential clinical uses in the treatment of fungal infections, and potential agronomic uses in the control of phytopathogenic diseases.

#### 3.3.3. Other Oxylipins

In addition to hydroxy and acetylenic fatty acids, other oxylipins have antifungal activity. The fatty acids (9E,11Z)-12-oxo-9,11-octadecadienoic acid and (10E,12E)-9-oxo-10,12-octadecadienoic acid were found to be active against a vast range of fungal plant pathogens in in vitro studies. These were particularly effective against *Phomopsis* species, which are responsible for small fruit blight, leaf spot, and rot [104].

Prost and co-workers (2005) tested 43 natural oxylipins against 13 plant pathogenic microorganisms, including five fungi and two oomycetes. Half of the compounds tested were strongly active, showing approximately 50% of growth inhibition in eukaryotic pathogens. These displayed different sensitivities to oxylipins. One of the most active oxylipins was 12-Oxo-10,15(Z)-phytodienoic acid (12-oxo-PDA), which is a well-known plant signal molecule in plant stress responses. Four PUFA hydroperoxides (13(*S*)-Hydroperoxy-9(*Z*),11(*E*),15(*Z*)-octadecatrienoic acid (13-HPOT), 9(S)-Hydroperoxy-10(E),12(Z),15(Z)-octadecatrienoic acid (9-HPOT), 9(S)-Hydroperoxy-10(E),12(Z)-octadecadienoic acid (9-HPOD), and 13(*S*)-Hydroperoxy-9(*Z*),11(*E*)-octadecadienoic acid (13-HPOD)), as well as their reduced forms (13(S)-Hydroxy-9(Z),11(E),15(Z)-octadecatrienoic acid (13-HOT), 9(*S*)-Hydroxy-10(*E*),12(*Z*),15(*Z*)-octadecatrienoic acid (9-HOT), 13(S)-Hydroxy-9(Z),11(E)-octadecadienoic acid (13S-HODE), and 9(*S*)-Hydroxy-10(*E*),12(*Z*)-octadecadienoic acid (9S-HODE)), and their dehydration products (ketotrienes, 13-Keto-9(*Z*),11(*E*),15(*Z*)-octadecatrienoic acid (13-KOT) and 9-Keto-10(E),12(Z),15(Z)-octadecatrienoic acid (9-KOT)) were also among the most active oxylipins [72]. Growth inhibition activity of pathogens seems to be extensive among oxylipins, with active compounds being found along the entire oxylipin pathway, which suggests that production of antimicrobial compounds is not separated from production of some plant signals.

However, the same authors observed that the inhibition effect of most of these oxylipins was transitory, with target microorganisms resuming growth in 48 to 72 h. Only 12-Oxo-PDA and 9(S),12(S),13(S)-Trihydroxy-10(E)-octadecenoic acid (9,12,13-THOE) were shown to be stable.

Similarly, Gráner and co-workers (2003) tested ten naturally occurring oxylipins as antifungals against pathogens interfering with *Brassica* cultivation and found inhibitory effects for several of them. Again, in some cases, the antifungal effect was overcome with time, which the authors theorize that it was due to degradation by the fungus or by acquired resistance [79].

It is currently accepted that divinyl esters, keto- and hydroxy-fatty acids, and hydroperoxides show strong antimicrobial activities, while volatile aldehydes were thought to only take part in signaling. Nevertheless, most works reporting antifungal ability of oxylipins are conducted in vitro, thus it is still unclear if oxylipins can have inhibitory activity in field conditions. In nature, several pathways control stress responses, and their interaction with each other makes difficult to predict oxylipins antimicrobial activity.

## 4. Fatty Acids as Mycotoxin Inhibitors

Mycotoxins are secondary metabolites produced by several species of filamentous fungi and can be highly toxic to humans and animals, compromising the quality and safety of the final product. Few authors have considered the impact of fatty acids in the production of mycotoxins. Still, some in vitro studies have indicated that fatty acids could either directly or indirectly affect mycotoxin contamination.

There are indications of a connection between host lipids and the ability of fungi to produce mycotoxins. For instance, *Aspergilli* usually grow and produce mycotoxins in oil-rich crops, such as corn, peanuts, cotton, and other nuts. Host lipids and oxylipins may act as signals to modulate fungal development, including the synthesis of mycotoxins, by mimicking the regulatory action of endogenous fungal oxidized lipids [105].

In addition, it is reported that in contaminated crop seeds with different lipid contents (peanut, sorghum, cowpea, and green gram), *A. parasiticus* produced higher amounts of aflatoxin B1 in the seed with highest lipid content (in this case peanut) [106]. Other studies prove this relation between lipid content of the substrate and mycotoxin synthesis. Mellon et al. (2000) found that cotton seed stored lipids supported the growth and aflatoxin production by *A. flavus*, when present as the sole carbon source, and that the removal of lipids from cottonseed caused an 800-fold drop in aflatoxin production [107]. Keller and co-workers (1994) found that in the presence of simple sugars, fungi hydrolyzed triglycerides, converting them into biomass and aflatoxin B1. Moreover, when invading nonwounded corn kernels, the fungus targets tissues where saccharides and triglycerides are present in highest concentrations [108]. Similarly, Rajasekaran et al. (2017) determined that aflatoxin production in cottonseed correlates with the level of lipids in the seed [109]. High oil hybrids of corn grains were found to be more susceptible to *Aspergillus* ear rot and aflatoxin production in grain [110], and fatty acids in maize have been reported to stimulate the β-oxidation pathway in the mitochondria and contribute to aflatoxin production [111]; however, the exact mechanism by which this stimulation occurs remains unclear. In contrast, other authors found mostly an inhibitory relationship between fatty acids and mycotoxin production. Nobili et al. (2019) tested the lipophilic extract of buckwheat hull, a grain rich in fatty acids, phenolic compounds, tocopherols, and phytosterols, to inhibit *A. flavus* and aflatoxin B1. Lipophilic extract showed at 10 μg/mL and average inhibition of over 80% of aflatoxin production and 74% of mycelial growth. However, no study was conducted to evaluate which component of the lipophilic fraction was responsible for the inhibitory activity [38].

Individual fatty acids and their derivatives were found to regulate fungal growth and production of mycotoxins; however, this action is dependent on the fatty acid structure and its concentration, making it difficult to predict the outcome. Aflatoxin B1 production by *A. parasiticus* has been found to be stimulated by a mix of fatty acids extracted from groundnut oil. Myristic, palmitic, and stearic acid increased aflatoxin B1 synthesis, while oleic and linoleic acids seem to suppress the toxin production [112]. Palmitic and stearic acids have been reported as supporters of aflatoxin production elsewhere [113,114]. Arachidic acid, a saturated fatty acid present in corn and peanut, has also been found to be related to elevated aflatoxin levels in these crops [115]. In synthetic media, lauric and palmitic acids were found to inhibited aflatoxin production (10–75%) in concentrations between 10 and 300 mM, while linoleic and linolenic acid increased massively (3400% and 1100%, respectively) the content of aflatoxin at 50 and 10 mM, respectively [116]. In addition, linolenic acid has been found to promote aflatoxin production. However, when autoxidized (exposed to air), it promoted mycelial growth and sporulation but inhibited the expression of aflatoxin synthesis gene clusters. Further analysis led to the identification of several oxylipin species [113].

Jasmonic acid has been thought as only taking part in signaling pathways, but more recently studies suggest that this compound may have antimicrobial activity. Orsoni et al. (2020) analyzed the interference of this oxylipin and its derivatives in mycotoxin biosynthesis. For T-2/HT-2 toxin (produced by *Fusarium sporotrichioides*), cis-jasmosne thiosemicarbazone (JTS) and dihydrojasmone thiosemicarbazone (Jdi) were effective, resulting in 40% inhibition at 5 μM and 75% at 25 μM concentration, respectively. For aflatoxins (produced by *A. flavus*), ketones had a less pronounced effect (30% at 100 μM), while with JTS a 98% inhibition was reached at the same concentration. Authors also found that Bis(dihydrojasmone thiosemicarbazone)copper(III) (JdiTS-Cu) impaired aflatoxin production by 70% (100 μM), targeting specifically the aflatoxin biosynthesis pathway, since it did not inhibit sclerotia development and hyphae growth, unlike the other compounds tested [117]. In addition, regarding oxylipins, 13S-hydroperoxy fatty acids at 1 to 100 μM suppressed aflatoxin and sterigmatocystin pathway gene expression, leading to significant reduction in toxin production of *A. parasiticus* and *Aspergillus nidulans*; however, 9S-HPODE promoted aflatoxin production [118]. It was found that *A. flavus* infection of peanut seed promotes linoleate 9-LOX expression, leading to the production of 9S-HPODE that stimulates aflatoxin production. On the other hand, *A. flavus* contamination repressed 13S-HPODE-producing LOX. Since both these oxylipins affect aflatoxin biosynthesis and induce sporulation, it is thought that they may have structural and biosynthetic similarities to endogenous *Aspergillus* oxylipins [119].

These studies suggest that 13S lipoxygenases could be used in in situ strategies to control mycotoxin levels in several crops. Table 2 lists several fatty acids that have been shown to influence mycotoxin production.

Alterations in fungal oxylipins pathway can also be a strategy to control fungal growth and mycotoxin synthesis. The deletion of the oxylipin generating enzyme genes (*ppoB* or *ppoC*) in *A. nidulans* led to an increased sterigmatocystin production; however, deletion of *ppoA* reduced this toxin production [125]. In *A. flavus*, the deletion of lipoxygenases gene (*Aflox1*) hindered aflatoxin biosynthesis; surprisingly, when the mutant strain was used to inoculate maize kernels, an increased aflatoxin production was observed [126]. In another example, the downregulation of oxygenase genes (*ppoA*, *ppoB*, *ppoC,* and *ppoD*) and a lipoxygenase gene (*loxA*) caused *A. flavus* to increase aflatoxin production and the alteration from asexual development to sclerotium production; this implies that compounds produced by PPO and LOX enzymes are important in mycotoxin synthesis and spore formation [127,128]. Furthermore, a mutation in the PPO (psi-producing oxygenases) orthologue in *Fusarium sporotrichioides* resulted in an inhibition of T-2 toxin production [129]. *A. ochraceus* lacking LOX produced low levels of 13S-HPODE, exhibited slower conidia formation, increased sclerotia production, and lowered OTA production [120]. These studies and others with similar conclusions indicate that it may be possible to control the synthesis of mycotoxin and fungal development by regulating the activity of PPO and LOX. However, these strategies of fungal control have only been tested in the laboratory and their applicability in complex environments is very limited and difficult to accomplish.

All these results imply a complex relationship between fatty acids, and in particular oxylipins, and mycotoxin biosynthesis. A specific oxylipin may possess different effects on different fungal species, and selection of oxylipins with a wide antifungal spectrum may be difficult. Nevertheless, these compounds could have potential for their application in foods for controlling fungal development and mycotoxin production.

## 5. Conclusions

The most relevant aspects of the inhibitory interaction of fatty acids and their derivatives towards fungal growth and mycotoxin synthesis were reviewed. The literature has been describing, for many decades, the antifungal capabilities of saturated, unsaturated, and derivatives of fatty acids, with more recent attention being shifted towards oxylipins and their importance in plant defense against fungi. Many different studies regarding fatty acids’ mode of action indicate that the cell membrane, targeted enzymes, and interference in metabolic pathways (in the case of mycotoxin production) are the main targets of these compounds, in respect of antifungal activity. Effects of fatty acids and their derivatives are highly dependent of their structure, their concentration, and the target fungal species, making it difficult to search for a solution that will fit into a more general approach in fungal control. For instance, the same compound may have an inhibitory effect in one fungal species and an inducing effect in other. Many antifungals and antimycotoxigenic studies with fatty acids have been conducted in vitro or in very controlled model systems, making it difficult to extrapolate results in complex food systems (for food applications) or in vivo (for medical applications). Stability of these compounds also needs to be further addressed, as well as efficient methods for their application, in food and medicine.

Still, fatty acids and their derivatives show great potential as antifungal compounds. These compounds can be considered environmentally friendly and safe (in the case of thoroughly studied fatty acids). For oxylipins, more studies are needed to ensure the lack of toxicity in all compounds. Study of oxylipin formation pathways can lead to new ways of controlling fungal growth (such as deletion or downregulation of target genes in fungi) and to the discovery of novel antifungal agents. Moreover, studies regarding the interactions between plant and fungi mediated by oxylipins could lead to the engineering of more resistance crop varieties, contributing to food security.

## Figures and Tables

**Table 1:** Hydroxy fatty acids with antifungal activity and their MIC against target fungi.

Hydroxy Fatty Acids	Target Fungi	MIC	Reference
2-hydroxydecanoic acid; 3-(*R*)-hydroxydecanoic acid; 3-hydroxy-5-*cis*-dodecenoic acid; 3-(*R*)-hydroxydodecanoic acid and 3-(*R*)-hydroxytetradecanoic acid	*A. fumigatus*, *A. nidulans*, *K. marxianus*, *P. commune*, *P. roqueforti*, *P. anomala,* and *R. mucilaginosa*	MIC of 10 to100 μg/mL for the racemic forms of the 3-OH fatty acids	Sjögren et al. (2003) [82]
3-hydroxydecanoic acid	*A. fumigatus*, *P. anomala,* and *P. roqueforti*	MIC 100 μg/mL	Broberg et al. (2007) [94]
9-HODE; 13-HODE; DOD; 9,10,13-TriHOME(11) and 9,12,13-TriHOME(10)	*A. uncinatum; A. niger; A.repens; C. albicans; M. phaesolina; P. chrysogenum; P. funiculosum; T. mentagrophites;* and *V. dahliae*	MIC of mono-, di-, and trihydroxy fatty acids produced by *Pseudomonas*: 32 μg/mL against *A. uncinatum*, *M. phaseolina*, *P. funiculosum,* and *V. dahliae;* 64 μg/mL against *T. mentagrophites*; 140 μg/mL against *A. niger*, *A. repens*, *P. chrysogenum*; 256 μg/mL against *C. albicans*	Martin-Arjol et al. (2010) [96]
coriolic and ricinoleic acid	*A. niger*, *M. plumbeus,* and *P. roqueforti*	MICs of 100 to 700 μg/mL for coriolic acid and 2400 μg/mL for ricinoleic acid	Black et al. (2013) [81]
13-HOE, 10-HOE, coriolic acid, ricinoleic acid	*A. niger* and *P. roqueforti*	MIC of 250 to 420 μg/mL against *A. niger* and 260 to 380 μg/mL against *P. roqueforti*	Chen et al. (2016) [97]
Coriolic acid, ricinoleic acid, 10-HOE, 13-HOE	*A. brasiliensis*, *A. clavatus*, *A. niger*, *C. albicans*, *C. humilis*, *C. valida*, *M. plumbeus*, *P. roqueforti*, *P. membranefaciens*, *P. orientalis*, *S. cerevisiae*, *S. unisporus*, *T. delbrueckii*, *W. anomalus,* and *Zygosaccharomyces* spp.	For coriolic acid, MIC of 70 to 670 μg/mL against filamentous fungi and of 4000 to ≥8000 μg/mL against yeasts. For mono hydroxy acids MIC from 290 to 500 μg/mL against *A. niger* and *P. roqueforti*	Liang et al. (2017) [80]
3-hydroxy-5-dodecenoic acid	*A. fumigatus*	MIC of 3-hydroxy-5-dodecenoic acid against *A. fumigatus* was 210 μg/mL	Mun et al. (2019) [98]
coriolic acid; dimorphecolic acid, ricinoleic acid, 10-OH C18:1, kamlolenic acid, 2-hydroxy linolenic acid, 2-hydroxy oleic acid	*A. niger*, *C. albicans*, *C. valida*, *P. membranaefaciens*, *P. roqueforti*, and *S. cerevisiae*	MIC from 230 to 1500 μg/mL against *A. niger*; 330 to ≥8000 μg/mL against *P. roqueforti*; 3000 to ≥8000 μg/mL against *C. albicans*; 3000 to ≥8000 μg/mL against *S. cerevisiae*; 2000 to ≥8000 μg/mL against *C. valida*; 830 to ≥8000 μg/mL against *P. membranaefaciens*	Liang et al. (2020) [85]

9-HODE: 9-hydroxy-10,12-octadecadienoic acid; 13-HODE: 13-hydroxy-9,11-octadecadienoic acid; DOD -7,10-dihydroxy-8E-octadecenoic acid; 9,10,13-THOE(11): 9,10,13-trihydroxy-11-octadecenoic acid; 9,12,13-THOE(10): 9,12,13-trihydroxy-10-octadecenoic acid; coriolic acid: 13-hydroxy-9,11-octadecadienoic acid; ricinoleic acid: 12-hydroxy-9-octadecenoic; 13-HOE: 13-hydroxy-9-octadecenoic acid; 10-HOE: 10-hydroxy-12-octadecenoic acid; dimorphecolic acid: 9-hydroxy-10,12-octadecadienoic acid; kamlolenic acid: 18-hydroxy-9E,11E,13E-octadecatrienoic acid.

**Table 2:** Fatty acids and derivatives with activity in mycotoxin production.

Fatty Acids and Derivatives	Target Fungi	Mycotoxin	Mycotoxin Production Variation	Concentrations Tested	Reference
myristic acid	*A. parasiticus*	Aflatoxin	promoted production of AFL	5 mM	Priyadarshini et al. (1980) [112]
palmitic acid			promoted production of AFL		
stearic acid			promoted production of AFL		
oleic acid			inhibited production of AFL		
linoleic acid			inhibited production of AFL		
linoleic acid peroxide	*A. parasiticus*	Aflatoxins	increased by at least 5000%	0.5 mg/mL	Fabbri et al. [120]
linoleic acid hydroperoxide			increased by at least 3000%		
lauric acid	*A. parasiticus*	Aflatoxins	decreased by 10% to 45%	50 to 300 mM	Tiwari et al. (1986) [116]
myristic acid			decreased by 14%	300 mM	
palmitic acid			decreased by 11% to 76%	10 to 300 mM	
docosanoic acid			increased by 290% to 540%	10 to 150 mM	
linoleic acid			increased by 3400%	50 mM	
linolenic acid			increased by 1100%	10 mM	
linoleic acid hydroperoxides	*A. flavus*	Aflatoxin	promoted production of AFL	non-specified	Hamid and Smith (1987) [114]
stearic acid			promoted production of AFL		
linoleic acid			inhibited production of AFL		
9S-HPODE	*A. parasiticus*	Aflatoxins	inhibited by ~30%	1to 100 μM	Burow et al. (1997) [118]
13S-HPODE			inhibited by ~40%	1 to 100 μM	
13S-HPOTE		Aflatoxin and NOR	inhibited AFL by 100% and NOR by 90%	100 μM	
13S-HPODE	*A. nidulans*	ST	inhibited by 52% to 81%	1 to 100 μM	
9S-HPODE	*A. flavus*	Aflatoxin	increased production	non-specified	Wilson et al. [121]
methyl jasmonate	*A. parasiticus*	Aflatoxin	promoted production	1 mM	Vergopoulou et al. [122]
9S-HPODE	*A. flavus*	Aflatoxins	increased production	non-specified	Tsitsigiannis et al. (2005) [119]
13S-HPODE			inhibited production		
methyl jasmonate	*A. parasiticus*	Aflatoxin	1 μM promoted AFL production and 10 mM totally inhibited AFL production	1 μM to 10 mM	Meimaroglou et al. [123]
9-HODE	*F. graminearum*	DON	promoted production	non-specified	Nobili et al. (2014) [124]
9-HPODE					
stearic acid	*A. flavus*	Aflatoxins	increased production	0.1, 0.75, and 1.25 mM	Yan et al. (2015) [113]
linolenic acid			inhibited production	1.25 and 5 mM	
*cis*-jasmone	*A. flavus*	Aflatoxin	inhibited production up to 30%	25 to 100 μM	Orsoni et al. (2020) [117]
JTS			inhibited by 55% to 100%		
Jdi			inhibited production up to 30%		
JdiTS			inhibited by 20% to 70%		
JTS-Cu			inhibited by 45% to 90%		
JdiTS-Cu			inhibited by 40% to 70%		
*cis*-jasmone	*F. sporotrichioides*	HT-2 toxin	no inhibition	5 to 25 μM	
JTS			inhibited by 40% to 75%		
Jdi			no inhibition		
JdiTS			inhibited by 25% to 75%		

## Data Availability

Not applicable.

## References

[1] Lunn J., Theobald H.E. (2006). The Health Effects of Dietary Unsaturated Fatty Acids. Nutr. Bull..

[2] Akoh C.C. (2017). Food Lipids: Chemistry, Nutrition, and Biotechnology.

[3] Saini R.K., Keum Y.-S. (2018). Omega-3 and Omega-6 Polyunsaturated Fatty Acids: Dietary Sources, Metabolism, and Significance—A Review. Life Sci..

[4] Gunstone F.D. (2012). Fatty Acid and Lipid Chemistry.

[5] De Carvalho C.C.C.R., Caramujo M.J. (2018). The Various Roles of Fatty Acids. Molecules.

[6] Mozaffarian D., Aro A., Willett W.C. (2009). Health Effects of Trans-Fatty Acids: Experimental and Observational Evidence. Eur. J Clin. Nutr..

[7] FDA (2013). Tentative Determination Regarding Partially Hydrogenated Oils. Fed.

[8] WHO (2018). WHO Plan to Eliminate Industrially-Produced Trans-Fatty Acids from Global Food Supply.

[9] EU (2019). Commission Regulation (EU) 2019/649 of 24 April 2019 Amending Annex III to Regulation (EC) No 1925/2006 of the European Parliament and of the Council as Regards Trans Fat, Other than Trans Fat Naturally Occurring in Fat of Animal Origin. Off. J. Eur. Union.

[10] Blée E. (2002). Impact of phyto-oxylipins in plant defense. Trends Plant Sci..

[11] Wyss O., Ludwig B.J., Joiner R.R. (1945). The fungistatic and fungicidal action of fatty acids and related compounds. Arch. Biochem..

[12] Ansari M.A., Asiri S.M.M., Alzohairy M.A., Alomary M.N., Almatroudi A., Khan F.A. (2021). Biofabricated fatty acids-capped silver nanoparticles as potential antibacterial, antifungal, antibiofilm and anticancer agents. Pharmaceuticals.

[13] Clitherow K.H., Binaljadm T.M., Hansen J., Spain S.G., Hatton P.V., Murdoch C. (2020). Medium-chain fatty acids released from polymeric electrospun patches inhibit *Candida albicans* growth and reduce the biofilm viability. ACS Biomater. Sci. Eng..

[14] Pohl C.H., Kock J.L.F., Thibane V.S., Méndez-Villas A. (2011). Antifungal Free Fatty Acids: A Review. Science Against Microbial Pathogens: Communicating Current Research and Technological Advances.

[15] Dix N.J., Webster J. (2012). Fungal Ecology.

[16] Hawksworth D.L., Lücking R. (2017). Fungal diversity revisited: 2.2 to 3.8 million species. Microbiol. Spectr..

[17] Brown G.D., Denning D.W., Levitz S.M. (2012). Tackling human fungal infections. Science.

[18] Gonçalves B., Ferreira C., Alves C.T., Henriques M., Azeredo J., Silva S. (2016). Vulvovaginal candidiasis: Epidemiology, microbiology and risk factors. Crit. Rev. Microbiol..

[19] Chen J., Varma A., Diaz M.R., Litvintseva A.P., Wollenberg K.K., Kwon-Chung K.J. (2008). *Cryptococcus neoformans* strains and infection in apparently immunocompetent patients, China. Emerg. Infect. Dis..

[20] Denning D.W. (1998). Invasive aspergillosis. Clin. Infect. Dis..

[21] Araiza J., Canseco P., Bonifaz A. (2006). Otomycosis: Clinical and mycological study of 97 cases. Rev. Laryngol. Otol. Rhinol..

[22] Sen M., Lahane S., Lahane T.P., Parekh R., Honavar S.G. (2021). *Mucor* in a viral land: A tale of two pathogens. Indian J. Ophthalmol..

[23] Schnurer J., Magnusson J. (2005). Antifungal lactic acid bacteria as biopreservatives. Trends Food Sci. Technol..

[24] Leyva-Salas M., Mounier J., Valence F., Coton M., Thierry A., Coton E. (2017). Antifungal microbial agents for food biopreservation—A review. Microorganisms.

[25] Gonçalves A., Palumbo R., Guimarães A., Gkrillas A., Dall’Asta C., Dorne J.-L., Battilani P., Venâncio A. (2020). The route of mycotoxins in the grape food chain. Am. J. Enol. Vitic..

[26] Palumbo R., Gonçalves A., Gkrillas A., Logrieco A., Dorne J.-L., Dall’Asta C., Venâncio A., Battilani P. (2020). Mycotoxins in maize: Mitigation actions, with a chain management approach. Phytopathol. Mediterr..

[27] Gerez C.L., Carbajo M.S., Rollan G., Torres Leal G., Font de Valdez G. (2010). Inhibition of citrus fungal pathogens by using lactic acid bacteria. J. Food Sci..

[28] Fisher M.C., Henk D.A., Briggs C.J., Brownstein J.S., Madoff L.C., McCraw S.L., Gurr S.J. (2012). Emerging fungal threats to animal, plant and ecosystem health. Nature.

[29] Guimarães A. (2019). Inhibition of Fungal Growth and Mycotoxin Production by Lactic Acid Bacteria.

[30] Jard G., Liboz T., Mathieu F., Guyonvarc’h A., Lebrihi A. (2011). Review of mycotoxin reduction in food and feed: From prevention in the field to detoxification by adsorption or transformation. Food Addit. Contam. Part A.

[31] Castillo N.I., Ibáñez M., Beltrán E., Rivera-Monroy J., Ochoa J.C., Páez-Castillo M., Posada-Buitrago M.L., Sulyok M., Hernández F. (2016). Identification of mycotoxins by UHPLC–QTOF MS in airborne fungi and fungi isolated from industrial paper and antique documents from the Archive of Bogotá. Environ. Res..

[32] Eskola M., Kos G., Elliott C.T., Hajšlová J., Mayar S., Krska R. (2020). Worldwide contamination of food-crops with mycotoxins: Validity of the widely cited ‘FAO estimate’ of 25%. Crit. Rev. Food Sci. Nutr..

[33] EC (2018). Commission Regulation (EC) No 1881/2006 of 19 December 2006 setting maximum levels for certain contaminants in foodstuffs. Off. J. Eur. Union.

[34] WHO/FAO (2016). Evaluation of Certain Food Additives and Contaminants: Eightieth Report of the Joint FAO/WHO Expert Committee on Food Additives.

[35] Bennett J.W., Klich M. (2003). Mycotoxins. Clin. Microbiol. Rev..

[36] Reis J.A., Paula A.T., Casarotti S.N., Penna A.L.B. (2012). Lactic acid bacteria antimicrobial compounds: Characteristics and applications. Food Eng. Rev..

[37] Altieri C., Cardillo D., Bevilacqua A., Sinigaglia M. (2007). Inhibition of *Aspergillus* spp. and *Penicillium* spp. by fatty acids and their monoglycerides. J. Food Prot..

[38] Nobili C., De Acutis A., Reverberi M., Bello C., Leone G.P., Palumbo D., Natella F., Procacci S., Zjalic S., Brunori A. (2019). Buckwheat hull extracts inhibit *Aspergillus flavus* growth and AFB1 biosynthesis. Front. Microbiol..

[39] Avis T.J., Bélanger R.R. (2001). Specificity and mode of action of the antifungal fatty acid cis-9-heptadecenoic acid produced by *Pseudozyma flocculosa*. Appl. Environ. Microbiol..

[40] Thibane V.S., Ells R., Hugo A., Albertyn J., Van Rensburg W.J.J., Van Wyk P.W., Kock J.L., Pohl C.H. (2012). Polyunsaturated fatty acids cause apoptosis in *C. albicans* and *C. dubliniensis* biofilms. Biochim. Biophys. Acta Gen. Subj..

[41] Suzuki K., Shono F., Kai H., Uno T., Uyeda M. (2000). Inhibition of topoisomerases by fatty acids. J. Enzym. Inhib..

[42] Yonezawa Y., Hada T., Uryu K., Tsuzuki T., Eitsuka T., Miyazawa T., Murakami-Nakai C., Yoshida H., Mizushina Y. (2005). Inhibitory effect of conjugated eicosapentaenoic acid on mammalian DNA polymerase and topoisomerase activities and human cancer cell proliferation. Biochem. Pharmacol..

[43] Pommier Y. (2006). Topoisomerase I inhibitors: Camptothecins and beyond. Nat. Rev. Cancer.

[44] Wood R., Lee T. (1981). Metabolism of 2-hexadecynoate and inhibition of fatty acid elongation. J. Biol. Chem..

[45] Xu T., Tripathi S.K., Feng Q., Lorenz M.C., Wright M.A., Jacob M.R., Mask M.M., Baerson S.R., Li X.-C., Clark A.M. (2012). A potent plant-derived antifungal acetylenic acid mediates its activity by interfering with fatty acid homeostasis. Antimicrob. Agents Chemother..

[46] Li X.-C., Jacob M.R., ElSohly H.N., Nagle D.G., Smillie T.J., Walker L.A., Clark A.M. (2003). Acetylenic acids inhibiting azole-resistant *Candida albicans* from *Pentagonia gigantifolia*. J. Nat. Prod..

[47] Parang K., Knaus E.E., Wiebe L.I., Sardari S., Daneshtalab M., Csizmadia F. (1996). Synthesis and antifungal activities of myristic acid analogs. Arch. Pharm..

[48] Branen A., Davidson P., Katz B. (1980). Antimicrobial properties of phenolic antioxidants and lipids. Food Technol..

[49] Kabara J., Vrable R., Lie Ken Jie M. (1977). Antimicrobial lipids: Natural and synthetic fatty acids and monoglycerides. Lipids.

[50] Bergsson G., Arnfinnsson J., Steingrímsson O., Thormar H. (2001). *In vitro* killing of *Candida albicans* by fatty acids and monoglycerides. Antimicrob. Agents Chemother..

[51] Lee J.H., Kim Y.G., Khadke S.K., Lee J. (2021). Antibiofilm and antifungal activities of medium-chain fatty acids against *Candida albicans* via mimicking of the quorum-sensing molecule farnesol. Microb. Biotechnol..

[52] Prasath K.G., Sethupathy S., Pandian S.K. (2019). Proteomic analysis uncovers the modulation of ergosterol, sphingolipid and oxidative stress pathway by myristic acid impeding biofilm and virulence in *Candida albicans*. J. Proteom..

[53] Muthamil S., Balasubramaniam B., Balamurugan K., Pandian S.K. (2018). Synergistic effect of quinic acid derived from *Syzygium cumini* and undecanoic acid against *Candida* spp. biofilm and virulence. Front. Microbiol..

[54] Bhattacharyya A., Sinha M., Singh H., Patel R.S., Ghosh S., Sardana K., Ghosh S., Sengupta S. (2020). Mechanistic insight into the antifungal effects of a fatty acid derivative against drug-resistant fungal infections. Front. Microbiol..

[55] Chadeganipour M., Haims A. (2001). Antifungal activities of pelargonic and capric acid on *Microsporum gypseum*. Mycoses.

[56] Liu S., Ruan W., Li J., Xu H., Wang J., Gao Y., Wang J. (2008). Biological control of phytopathogenic fungi by fatty acids. Mycopathologia.

[57] Aneja M., Gianfagna T.J., Hebbar P.K. (2005). *Trichoderma harzianum* produces nonanoic acid, an inhibitor of spore germination and mycelial growth of two cacao pathogens. Physiol. Mol. Plant Pathol..

[58] Lafon-Lafourcade S., Geneix C., Ribéreau-Gayon P. (1984). Inhibition of alcoholic fermentation of grape must by fatty acids produced by yeasts and their elimination by yeast ghosts. Appl. Environ. Microbiol..

[59] Altieri C., Bevilacqua A., Cardillo D., Sinigaglia M. (2009). Antifungal activity of fatty acids and their monoglycerides against *Fusarium* spp. in a laboratory medium. Int. J. Food Sci..

[60] Corsetti A., Gobbetti M., Rossi J., Damiani P. (1998). Antimould activity of sourdough lactic acid bacteria: Identification of a mixture of organic acids produced by *Lactobacillus sanfrancisco* CB1. Appl. Microbiol. Biotechnol..

[61] Desbois A.P., Smith V.J. (2010). Antibacterial free fatty acids: Activities, mechanisms of action and biotechnological potential. Appl. Microbiol. Biotechnol..

[62] Kabara J.J., Swieczkowski D.M., Conley A.J., Truant J.P. (1972). Fatty acids and derivatives as antimicrobial agents. Antimicrob. Agents Chemother..

[63] Wood J., Richardson R., Nute G., Fisher A., Campo M., Kasapidou E., Sheard P., Enser M. (2004). Effects of fatty acids on meat quality: A review. Meat. Sci..

[64] Benyagoub M., Willemot C., Bélanger R. (1996). Influence of a subinhibitory dose of antifungal atty acids from *Sporothrix flocculosa* on cellular lipid composition in fungi. Lipids.

[65] Thibane V.S., Kock J.L., Ells R., Van Wyk P.W., Pohl C.H. (2010). Effect of marine polyunsaturated fatty acids on biofilm formation of *Candida albicans* and *Candida dubliniensis*. Mar. Drugs.

[66] Walters D., Raynor L., Mitchell A., Walker R., Walker K. (2004). Antifungal activities of four fatty acids against plant pathogenic fungi. Mycopathologia.

[67] Calvo A.M., Hinze L.L., Gardner H.W., Keller N.P. (1999). Sporogenic effect of polyunsaturated fatty acids on development of *Aspergillus* spp.. Appl. Environ. Microbiol..

[68] Madi L., Wang X., Kobiler I., Lichter A., Prusky D. (2003). Stress on avocado fruits regulates Δ9-stearoyl ACP desaturase expression, fatty acid composition, antifungal diene level and resistance to *Colletotrichum gloeosporioides* attack. Physiol. Mol. Plant Pathol..

[69] Deboever E., Deleu M., Mongrand S., Lins L., Fauconnier M.-L. (2020). Plant–pathogen interactions: Underestimated roles of phyto-oxylipins. Trends Plant Sci..

[70] Tsitsigiannis D.I., Keller N.P. (2007). Oxylipins as developmental and host–fungal communication signals. Trends Microbiol..

[71] Christensen S.A., Kolomiets M.V. (2011). The lipid language of plant–fungal interactions. Fungal Genet. Biol..

[72] Prost I., Dhondt S., Rothe G., Vicente J., Rodriguez M.J., Kift N., Carbonne F., Griffiths G., Esquerré-Tugayé M.-T., Rosahl S. (2005). Evaluation of the antimicrobial activities of plant oxylipins supports their involvement in defense against pathogens. Plant Physiol..

[73] Mehmood A., Liu G., Wang X., Meng G., Wang C., Liu Y. (2019). Fungal quorum-sensing molecules and inhibitors with potential antifungal activity: A review. Molecules.

[74] Rancé I., Fournier J., Esquerré-Tugayé M.-T. (1998). The incompatible interaction between Phytophthora parasitica var. nicotianae race 0 and tobacco is suppressed in transgenic plants expressing antisense lipoxygenase sequences. Proc. Natl. Acad. Sci. USA.

[75] Mène-Saffrané L., Esquerré-Tugayé M.-T., Fournier J. (2003). Constitutive expression of an inducible lipoxygenase in transgenic tobacco decreases susceptibility to Phytophthora parasitica var. nicotianae. Mol. Breed..

[76] Seo H.S., Song J.T., Cheong J.-J., Lee Y.-H., Lee Y.-W., Hwang I., Lee J.S., Do Choi Y. (2001). Jasmonic acid carboxyl methyltransferase: A key enzyme for jasmonate-regulated plant responses. Proc. Natl. Acad. Sci. USA.

[77] Vigor C., Bertrand-Michel J., Pinot E., Oger C., Vercauteren J., Le Faouder P., Galano J.-M., Lee J.C.-Y., Durand T. (2014). Non-enzymatic lipid oxidation products in biological systems: Assessment of the metabolites from polyunsaturated fatty acids. J. Chromatogr. B.

[78] Eckardt N.A. (2008). Oxylipin signaling in plant stress responses. Plant Cell.

[79] Granér G., Persson P., Meijer J., Alström S. (2003). A study on microbial diversity in different cultivars of *Brassica napus* in relation to its wilt pathogen, *Verticillium longisporum*. FEMS Microbiol. Lett..

[80] Liang N., Cai P., Wu D., Pan Y., Curtis J.M., Ganzle M.G. (2017). High-speed counter-current chromatography (HSCCC) purification of antifungal hydroxy unsaturated fatty acids from plant-seed oil and *Lactobacillus* cultures. J. Agric. Food Chem..

[81] Black B.A., Zannini E., Curtis J.M., Gänzle M.G. (2013). Antifungal hydroxy fatty acids produced during sourdough fermentation: Microbial and enzymatic pathways, and antifungal activity in bread. Appl. Environ. Microbiol..

[82] Sjögren J.R., Magnusson J., Broberg A., Schnürer J., Kenne L. (2003). Antifungal 3-hydroxy fatty acids from *Lactobacillus plantarum* MiLAB 14. Appl. Environ. Microbiol..

[83] Hou C., Iii R.F. (2000). Growth inhibition of plant pathogenic fungi by hydroxy fatty acids. J. Ind. Microbiol. Biotechnol..

[84] Weber H. (2002). Fatty acid-derived signals in plants. Trends Plant Sci..

[85] Liang N. (2020). Hydroxy Fatty Acids: Structures and Antifungal Activities in Foods.

[86] Kato T., Nakai T., Ishikawa R., Karasawa A., Namai T. (2001). Preparation of the enantiomers of hydroxy-C18 fatty acids and their anti-rice blast fungus activities. Tetrahedron Asymmetry.

[87] Pohl E.E., Voltchenko A.M., Rupprecht A. (2008). Flip-flop of hydroxy fatty acids across the membrane as monitored by proton-sensitive microelectrodes. Biochim. Biophys. Acta.

[88] Ek-von Mentzer B.A., Zhang F., Hamilton J.A. (2001). Binding of 13-HODE and 15-HETE to phospholipid bilayers, albumin, and intracellular fatty acid binding proteins: Implications for transmembrane and intracellular transport and for protection from lipid peroxidation. J. Biol. Chem..

[89] Griffiths W.J., Wang Y. (2020). Lipidomics: Current and Emerging Techniques.

[90] Hartmann M.-A. (1998). Plant sterols and the membrane environment. Trends Plant Sci..

[91] Yasari A., Liang N., Foroutan A., Gänzle M.G., Strelkov S.E., Kav N.N. (2020). Investigating the potential of unsaturated fatty acids as antifungal crop protective agents. Can. J. Plant Sci..

[92] Liang N., Dacko A., Tan A.K., Xiang S., Curtis J.M., Gänzle M.G. (2020). Structure-function relationships of antifungal monohydroxy unsaturated fatty acids (HUFA) of plant and bacterial origin. Food Res. Int..

[93] Ndagano D., Lamoureux T., Dortu C., Vandermoten S., Thonart P. (2011). Antifungal activity of two lactic acid bacteria of the *Weissella* genus isolated from food. J. Food Sci..

[94] Broberg A., Jacobsson K., Strom K., Schnurer J. (2007). Metabolite profiles of lactic acid bacteria in grass silage. Appl. Environ. Microbiol..

[95] Brosnan B., Coffey A., Arendt E.K., Furey A. (2012). Rapid identification, by use of the LTQ Orbitrap hybrid FT mass spectrometer, of antifungal compounds produced by lactic acid bacteria. Anal. Bioanal. Chem..

[96] Martin-Arjol I., Bassas-Galia M., Bermudo E., Garcia F., Manresa A. (2010). Identification of oxylipins with antifungal activity by LC–MS/MS from the supernatant of *Pseudomonas* 42A2. Chem. Phys. Lipids.

[97] Chen Y.Y., Liang N.Y., Curtis J.M., Gänzle M.G. (2016). Characterization of linoleate 10-hydratase of *Lactobacillus plantarum* and novel antifungal metabolites. Front. Microbiol..

[98] Mun S.Y., Kim S.K., Woo E.R., Chang H.C. (2019). Purification and characterization of an antimicrobial compound produced by *Lactobacillus plantarum* EM showing both antifungal and antibacterial activities. LWT.

[99] Gershon H., Shanks L. (1978). Antifungal properties of 2-alkynoic acids and their methyl esters. Can. J. Microbiol..

[100] Carballeira N. (2008). New advances in fatty acids as antimalarial, antimycobacterial and antifungal agents. Prog. Lipid Res..

[101] Carballeira N.M., Sanabria D., Parang K. (2005). Total synthesis and further scrutiny of the *in vitro* antifungal activity of 6-nonadecynoic acid. Arch. Pharm..

[102] Carballeira N.M., Sanabria D., Cruz C., Parang K., Wan B., Franzblau S. (2006). 2, 6-Hexadecadiynoic acid and 2, 6-nonadecadiynoic acid: Novel synthesized acetylenic fatty acids as potent antifungal agents. Lipids.

[103] Yoon M.Y., Choi G., Choi Y., Jang K., Park M., Cha B., Kim J.C. (2010). Effect of polyacetylenic acids from *Prunella vulgaris* on various plant pathogens. Lett. Appl. Microbiol..

[104] Cantrell C.L., Case B.P., Mena E.E., Kniffin T.M., Duke S.O., Wedge D.E. (2008). Isolation and identification of antifungal fatty acids from the basidiomycete *Gomphus floccosus*. J. Agric. Food Chem..

[105] Gao X., Kolomiets M.V. (2009). Host-derived lipids and oxylipins are crucial signals in modulating mycotoxin production by fungi. Toxin Rev..

[106] Reddy M.J., Shetty H.S., Fanelli C., Lacey J. (1992). Role of seed lipids in *Aspergillus parasiticus* growth and aflatoxin production. J. Sci. Food Agric..

[107] Mellon J.E., Cotty P.J., Dowd M.K. (2000). Influence of lipids with and without other cottonseed reserve materials on aflatoxin B1 production by *Aspergillus flavus*. J. Agric. Food Chem..

[108] Keller N.P., Butchko R.A., Sarr B., Phillips T.D. (1994). A visual pattern of mycotoxin production in maize kernels by *Aspergillus* spp.. Phytopathology.

[109] Rajasekaran K., Ford G., Sethumadhavan K., Carter-Wientjes C., Bland J., Cao H., Bhatnagar D. (2017). *Aspergillus flavus* growth and aflatoxin production as influenced by total lipid content during growth and development of cottonseed. J. Crop. Improv..

[110] Severns D.E., Clements M.J., Lambert R.J., White D.G. (2003). Comparison of *Aspergillus* ear rot and aflatoxin contamination in grain of high-oil and normal-oil corn hybrids. J. Food Prot..

[111] Falade T., Chrysanthopoulos P.K., Hodson M.P., Sultanbawa Y., Fletcher M., Darnell R., Korie S., Fox G. (2018). Metabolites identified during varied doses of *Aspergillus* species in Zea mays grains, and their correlation with aflatoxin levels. Toxins.

[112] Priyadarshini E., Tulpule P. (1980). Effect of free fatty acids on aflatoxin production in a synthetic medium. Food Cosmet. Toxicol..

[113] Yan S., Liang Y., Zhang J., Chen Z., Liu C.-M. (2015). Autoxidated linolenic acid inhibits aflatoxin biosynthesis in *Aspergillus flavus* via oxylipin species. Fungal Genet. Biol..

[114] Hamid A.B., Smith J.E. (1987). Effect of exogenous lipids on growth and aflatoxin production by *Aspergillus flavus*. Trans. Br. Mycol. Soc..

[115] Canavar Ö., Kaynak M.A. (2013). Prevention of pre-harvest aflatoxin production and the effect of different harvest times on peanut (*Arachis hypogaea* L.) fatty acids. Food Addit. Contam. Part A.

[116] Tiwari R., Mittal V., Singh G., Bhalla T., Saini S., Vadehra D. (1986). Effect of fatty acids on aflatoxin production by *Aspergillus parasiticus*. Folia Microbiol..

[117] Orsoni N., Degola F., Nerva L., Bisceglie F., Spadola G., Chitarra W., Terzi V., Delbono S., Ghizzoni R., Morcia C. (2020). Double gamers—Can modified natural regulators of higher plants act as antagonists against phytopathogens? The case of jasmonic acid derivatives. Int. J. Mol. Sci..

[118] Burow G., Nesbitt T., Dunlap J., Keller N. (1997). Seed lipoxygenase products modulate *Aspergillus* mycotoxin biosynthesis. Mol. Plant-Microbe Interact..

[119] Tsitsigiannis D.I., Kunze S., Willis D.K., Feussner I., Keller N.P. (2005). *Aspergillus* infection inhibits the expression of peanut 13 S-HPODE-forming seed lipoxygenases. Mol. Plant-Microbe Interact..

[120] Fabbri A., Fanelli C., Panfili G., Passi S., Fasella P. (1983). Lipoperoxidation and aflatoxin biosynthesis by *Aspergillus parasiticus* and *A. flavus*. Microbiology.

[121] Wilson R.A., Gardner H.W., Keller N.P. (2001). Cultivar-dependent expression of a maize lipoxygenase responsive to seed infesting fungi. Mol. Plant-Microbe Interact..

[122] Vergopoulou S., Galanopoulou D., Markaki P. (2001). Methyl jasmonate stimulates aflatoxin B1 biosynthesis by *Aspergillus parasiticus*. J. Agric. Food Chem..

[123] Meimaroglou D.M., Galanopoulou D., Markaki P. (2009). Study of the effect of methyl jasmonate concentration on aflatoxin biosynthesis by *Aspergillus parasiticus* in yeast extract sucrose medium. Int. J. Microbiol..

[124] Nobili C., D’Angeli S., Altamura M.M., Scala V., Fabbri A.A., Reverberi M., Fanelli C. (2014). ROS and 9-oxylipins are correlated with deoxynivalenol accumulation in the germinating caryopses of *Triticum aestivum* after *Fusarium graminearum* infection. Eur. J. Plant Pathol..

[125] Tsitsigiannis D.I., Keller N.P. (2006). Oxylipins act as determinants of natural product biosynthesis and seed colonization in *Aspergillus nidulans*. Mol. Microbiol..

[126] Scarpari M., Bello C., Pietricola C., Zaccaria M., Bertocchi L., Angelucci A., Ricciardi M.R., Scala V., Parroni A., Fabbri A.A. (2014). Aflatoxin control in maize by *Trametes versicolor*. Toxins.

[127] Brown H.S., Scott J.B., Bhaheetharan J., Sharpee W.C., Milde L., Wilson R.A., Keller N.P. (2009). Oxygenase coordination is required for morphological transition and the host–fungus interaction of *Aspergillus flavus*. Mol. Plant-Microbe Interact..

[128] Brown H.S., Zarnowski R., Sharpee W., Keller N. (2008). Morphological transitions governed by density dependence and lipoxygenase activity in *Aspergillus flavus*. Appl. Environ. Microbiol..

[129] McDonald T., Devi T., Shimizu K., Sim S., Keller N. (2004). Signaling events connecting mycotoxin biosynthesis and sporulation in *Aspergillus* and *Fusarium* spp.. JSM Mycotoxins.

